# Postoperative Dressing Regimens in Nail Surgeries: A Scoping Review

**DOI:** 10.1002/jfa2.70100

**Published:** 2026-01-21

**Authors:** David Yousry Foad Isaac Wassef, Zainab Al‐Modhefer, Kym Hennessy

**Affiliations:** ^1^ School of Health Sciences Western Sydney University Campbelltown New South Wales Australia; ^2^ Translational Health Research Institute Western Sydney University Campbelltown New South Wales Australia

**Keywords:** dressings, nail surgeries, partial nail avulsions, total nail avulsions

## Abstract

**Background:**

Toenail surgeries are one of the most common surgeries performed on the lower limb with many common postoperative complications, including prolonged healing time, infection and prolonged exudation. There is a wide variety of dressings designed to reduce the rate of postoperative complications and improve healing. The efficacy of dressings for improving postoperative healing in toenail surgeries is still unknown. There is no consensus on the correct dressing regimen and a low number of studies into the subject. This review aimed to assess the current literature to determine the most efficacious postoperative dressing regimens following toenail surgeries.

**Methods:**

Searches were conducted in MEDLINE, Embase, CINAHL, Cochrane library and SCOPUS from database creation to June 2025. Quantitative studies which applied any postoperative dressing regimen to any patient undergoing any form of toenail surgery and attributed the postoperative outcome to the dressing were deemed eligible for inclusion. Two independent reviewers performed the study selection, data extraction and risk of bias assessment. Risk of bias was assessed using the JBI critical appraisal tools.

**Results:**

Fourteen studies met the inclusion criteria. The included studies had 27 dressing regimens across 15 outcome measures. Outcome measures investigated were as follows: pain, healing rate, time of exudate, inflammation, infection rate, bleeding, size of exudate, type of exudate, soothing effect, nail area preservation, time until normal shoe wear, satisfaction rate, time until full function, ease of removal by dressing type and ease of dressing removal by surgery type. Overall, no outcome measure had high quality evidence supporting use, suggesting no dressing was superior to others for any of the outcome measures.

**Conclusions:**

Collation of similar outcome measures was not possible due to the variability in outcome measures, and a lack of high‐quality studies investigating the efficacy of specific dressing regimens for the reduction of postoperative complications or improving postoperative healing meant specific conclusions could not be formed. However, a consensus in the included articles was that primary dressings were more likely to impact postoperative outcomes when compared to secondary dressings. Further high‐quality research investigating the efficacy of postoperative dressing regimens in nail surgery are needed.

## 1 Introduction

Nail surgeries are the most common type of surgery performed on the lower limb with onychocryptosis being the most common indication for nail surgery with an estimated prevalence of 0.46%–2.45% in the general population [1]. If left untreated, onychocryptosis can lead to infection, painful ambulation and decreased quality of life [2].

There are two main categories of nail surgeries: sharp excisional surgeries and matrix cauterisation surgeries. Sharp excisional surgeries typically close the wound and therefore create a wound that heals by primary intention which contrasts with wounds created in matrix cauterisation surgeries that heal by secondary intention [3]. The type of cautery agent selected also creates a unique wound environment, with acidic cautery agents causing coagulative necrosis [4, 5] and alkali agents causing liquefactive necrosis [6].

There are a number of postoperative complications that can occur following nail surgeries with infection being the most frequent at 4.2% which is 2.7 times greater rate than other surgeries of the lower limb [1]. Dressings may be an important tool in reducing rates of complications in these surgeries. Postoperative dressing regimens are designed to improve postoperative healing [7] and reduce the rate of postoperative complications [8]. Postoperative dressing regimens serve to cover the wound, modulate the physiological wound environment including moisture levels, reduce bioburden or infuse the wound with beneficial compounds [9]. To effectively utilise the advantages provided by a dressing, the dressing must be matched to the appropriate wound type [8].

Nail surgeries are particularly difficult in this respect due to the plethora of available surgical techniques, cautery agents and dressing types [10]. The last systematic review assessing the efficacy of postoperative dressings following toenail surgeries found no difference in the dressings assessed and only considered dressing to be a secondary outcome [11]. The most recent systematic review that assessed postoperative complications in toenail surgeries was a series by Exley et al. [12] and Exley et al. [13]. However, the efficacy of dressing regimens on postoperative healing was not assessed in these reviews. The benefits of dressings on wound healing are well established in high‐risk foot care, yet there is a distinct lack of reporting of postoperative dressing protocols in toenail surgery.

With numerous dressings available and the many claimed benefits provided by dressing manufacturers, it can be difficult to separate the marketing claims from the clinical efficacy of the dressings. Therefore, this scoping review aimed to determine efficacy of dressing regimens for postoperative healing and reducing complications of toenail surgeries.

## 2 Materials and Methods

### 2.1 Review Protocol

The Preferred Reporting Items for Systematic Reviews and Meta‐Analyses Scoping Review extension (PRISMA‐ScR) was used to ensure the minimum reporting standards for a scoping review were met [14].

### 2.2 Search Strategy

Medline, Embase, CINAHL, SCOPUS and Cochrane Library databases were searched from database inception until June 2025. The following search terms ‘nail diseases’, ‘nail surgery’, ‘postoperative care’ and ‘dressings’ and related synonyms were used to develop a comprehensive pragmatic search strategy. Standard MeSH terms were utilised where possible or an appropriate text word was adopted. Boolean operators were also used. The full detailed search strategy is available in Supporting Information S1: Additional File A. Additionally, reference lists of identified studies were hand searched for any additional articles.

### 2.3 Study Selection Criteria

Study designs eligible for inclusion in this review were randomised control trials (RCTs), quasi‐experimental studies, nonrandomised studies, case–control studies, cohort studies and case series. Single cases and reviews articles were excluded. Articles included were limited to English due to the language limitations of the reviewers. No restriction was placed on age of included articles to capture any articles that may have been indexed online since previous reviews.

Articles considered for this review were any that assessed the efficacy of any postoperative dressing regimen on any patient who had undergone any type of toenail surgery. For this scoping review, postoperative dressing regimens were deemed to be any dressing, topical medication or other postoperative protocol which was designed to interact with the wound surface and/or affect the physiological wound environment for a specified outcome. All quantitative outcomes attributed to postoperative dressing regimens were considered.

Titles and abstracts were independently screened by two reviewers (D.W. and Z.A.). Disagreements were settled by discussion or, if necessary, a third reviewer (K.H.). Full‐text articles meeting initial criteria were then assessed independently by two reviewers (D.W. and K.H.), with any unresolved conflicts adjudicated by the third reviewer (Z.A.).

### 2.4 Quality Assessment

Two reviewers (D.W. and K.H.) independently assessed risk of bias with the Joanna Briggs Institute (JBI) tools for RCTs [15], quasi‐experimental studies [16] and case series [17], with results recorded in Microsoft Word (Microsoft Corporation). Disagreements were discussed and, if unresolved, adjudicated by a third reviewer (Z.A.). The JBI risk of bias tools do not provide thresholds for classifying risk of bias into distinct categories such as high or low risk of bias. In this review, to grade the overall risk of bias for an individual outcome measure the number of ‘yes’ responses were divided by the number of questions for the outcome measure. This value was given as a percentage, with scores 75% and over deemed low risk of bias, scores between 50% and 74% being considered medium risk of bias and those below 50% being high risk of bias. These thresholds were an amalgamation of those previously used by Alfaleh et al. [18], Alluhaybi et al. [19] and Franco et al. [20].

### 2.5 Data Extraction

Data extraction was performed by two reviewers (D.W. and K.H.) and collated using Microsoft Excel (Microsoft Corporation). The following data were extracted from the included articles: participant description, participant attrition, follow‐up period, type of surgery, intervention, outcome measure, results and overall conclusion.

## 3 Results

### 3.1 Search Results

**FIGURE 1:**
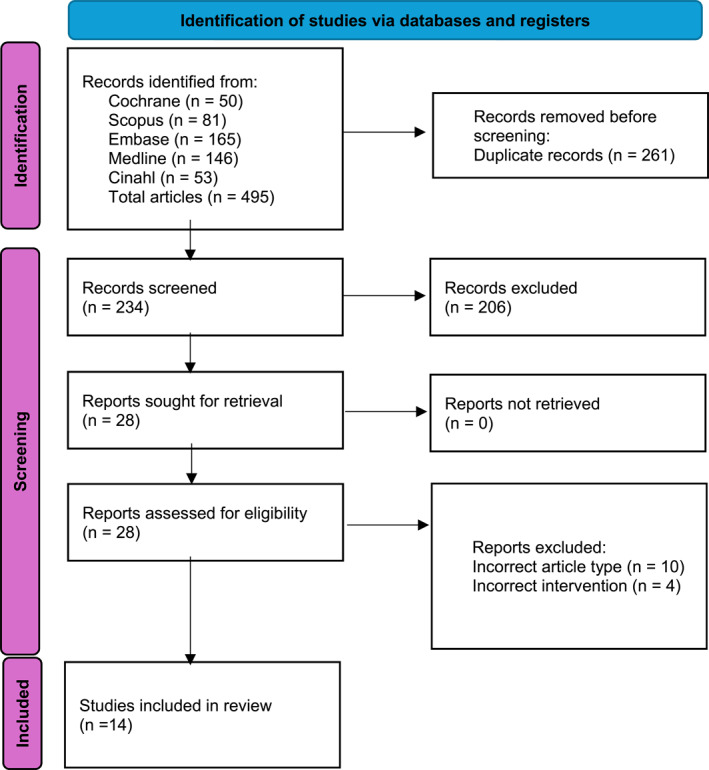
PRISMA flow diagram. PRISMA flow diagram adapted from Page et al. [21].

A total of 495 articles were retrieved using the detailed search strategy (Figure 1). The inclusion criteria were met by 14 studies, and each study is described in Table 1. The majority of included studies (11/14) were RCTs [4, 25, 26, 27, 28, 29, 30, 31, 32, 33, 34], with 2 quasi‐experimental studies [22, 23] and 1 case–control series [24].

**TABLE 1:** Description of studies.

Author (year)	Study type	Patient description	Participants starting/finishing	Follow‐up period	Surgery type	Intervention (no.)	Outcomes measured
Aksakal et al. [22]	Quasi‐experimental	Patients with stage III onychocryptosis	67/67	2–120 months	Phenol matrixectomy	Ferric chloride (32) Dry sterile sponge (25)	Exudate (days)
Altman et al. [23]	Quasi‐experimental	Not stated	U/231	4 weeks	Phenol matrixectomy	Silver sulfadiazine cream (66) 1% hydrocortisone (20) Silver sulfadiazine and 1% hydrocortisone cream (106) No topical medication (39)	Pain (y/n) Drainage (y/n) Inflammation (y/n) Infection (y/n).
Barreiro et al. [24]	Case series	Any patient who presented with onychocryptosis	60/54	4 weeks	Phenol matrixectomy	Oakin hydrogel (54)	Time to heal (weeks)
Bernardshaw et al. [25]	RCT	Patients ages 16 or over who received treatment for onychocryptosis	138/104	2 weeks	Phenol matrixectomy and surgical nail avulsions	Alkali baths (32) Acidic baths (34) No bath (31)	Pain (both VAS and NRS) Function of toe (y/n) Infection (y/n) Patient's perception on soothing (y/n)
Cordoba‐Fernandez and Lobo‐Martin [26]	RCT	Any patient with onychocryptosis	80/74	Minimum of 8 months	Winograd then phenol matrixectomy	Control: 20 halluces Conventional gelatin sponges: 25 halluces High porosity gelatin sponges: 29 halluces	Postsurgical blood loss (g) Digit circumference (Cm) Pain (VAS) Recovery time (days)
Cordoba‐Fernandez et al. [27]	Within patient RCT	Patients presenting with stage I or IIa (Mozena classification) onychocryptosis on their halluces	70/70	Until healed (16 days was maximum)	Suppan 1	Autologous platelets (35) Nitrofurazone ointment (35)	Pain on day 3 (VAS) Healing time (days) Inflammation (cm)
Dovison and Keenan [28]	RCT	Patients who were stated to be ‘Healthy’	42/36	Until healed (maximum was 46 days)	Phenol matrixectomy	Paraffin gauze (13) Povidone iodine (13) Hydrogel (16)	Healing time (days)
Drago et al. [29]	RCT	Patients who required isolated nail surgery	44/37	Until healed (42 days longest)	Phenol matrixectomy	Dextran (14) Otic cortisporin (6) Silvadene (6) Domeboro (3) Saline (2) Neosporin powder (5)	Pain (scale of 0–3) Diameter of exudate (mm) Exudate duration (days)
Foley and Allen [4]	RCT	Patients referred by their general practitioner for toenail surgery	U/70	Until healed (70 days maximum)	Phenol matrixectomy	Calcium alginate (35) Low adherent dressing (35)	Healing time (days)
Lopezosa‐Reca et al. [30]	RCT	Patients between 14 and 40 with Kline classification stage I, II or III or Mozena classification stage I or IIa onychocryptosis	70/70	20 days	Phenol matrixectomy	1 mL of 2% hyaluronic acid (35) 1 mL of povidone iodine gel (35)	Healing time (days) Inflammation (y/n) Pain (VAS) Exudate (y/n)
McIntosh and Thomson [31]	RCT	Patients over 16 who were deemed suitable for toenail surgery	100/87	Until complete wound healing.	Phenol matrixectomy	Honey dressings (52) Paraffin tulle gras (48)	Healing time (days) Pain (VAS)
Oliveira et al. [32]	RCT	Patients who require nail plate avulsion	26/26	180 days	Surgical nail avulsions	Petroleum jelly and gauze (11) Cellulose nail (15)	Pain (VAS) Area (mm^2^) Patient satisfaction (%)
Sykes et al. [33]	RCT	Ingrown and onychogryphotic nails	274/185	2 days	Phenol matrixectomy, Zadik and surgical nail avulsion	Light paraffin (93) Heavy paraffin (93) Low adherent dressing (88)	Easy of dressing removal (very easy/easy/medium/difficult/very difficult)
van Gils et al. [34]	RCT	Patients with infected or chronically ingrown toenails	20/19	8 weeks	Sodium hydroxide matrixectomy	Collagen alginate (9) Sulfadiazine silver cream and gauze (10)	Healing time (days)

Abbreviations: *n*, no; NRS, numeric rating scale; RCT, randomised control trial; U, unknown; VAS, visual analogue scale; y, yes.

### 3.2 Summary of the Description of Studies

From the included studies, 27 postoperative dressing regimens were identified. These were as follows: oakin hydrogel, hydrogel, nitrofurazone ointment, gauze, autologous platelets, gelatin sponges, povidone iodine, calcium alginate, nonadherent dressings, paraffin gauze, hyaluronic acid, honey dressings, sulfadiazine silver, collagen alginate, Burow's solution, hydrocortisone cream, acidic soap, alkaline soap, otic corticosporin, Silvadene, Domeboro, saline, Neosporin powder, dextran, cellulose nail substitute, petroleum jelly and ferric chloride.

Included articles utilised a range of surgical techniques including phenol matrixectomies [11/14] [4, 22, 23, 24, 25, 26, 28, 29, 30, 31, 33], sodium hydroxide matrixectomies [1/14] [34], Zadik procedures [1/14] [33], Suppan I [1/14] [27] and simple nail avulsions [3/14] [25, 32, 33]. The number of participants in the included articles ranged from 19 to 104 participants. A full summary of the study characteristics is presented in Table 1.

### 3.3 Quality Appraisal

The risk of bias of included articles ranged from high to low. Out of the 14 articles, eight were categorised as high risk of bias which included five RCTs [4, 28, 29, 33, 34], two quasi‐experimental studies [22, 23] and one case series [24]. Four RCTS had medium risk of bias [25, 30, 31, 32], and two RCTs had low risk of bias [26, 27]. Notably, the domain with lowest ratio of ‘yes’ answers was administration of the intervention or exposure, with 8/14 papers scoring 33.3% or less for the domain meaning there is a higher chance of performance bias in those papers. The full risk of bias assessments are presented in Supporting Information S1: Additional File B for case series, quasi‐experimental and RCTs, respectively.

### 3.4 Outcomes

Overall, this review found 15 unique outcome measures in the 14 included articles. A full summary can be found in Table 2.

**TABLE 2:** Summary of study results.

Domain	Author (year)	Control (no.)	Intervention (no.)	Results (mean ± SD unless specified otherwise)	Risk of bias for article
Healing rate	Barreiro et al. [24]	N/A	Oakin hydrogel (54)	Number of patients that achieved full healing (per week) 43/54 (80%) of patients healed in the first week 6/54 (11%) healed in the second week 4/54 (7%) healed in the third week (No significance values given)	High
	Cordoba‐Fernandez et al. [27]	Nitrofurazone ointment (35)	Autologous platelets (35)	Time for full healing (days) Control: 12.3 ± 1.99 days Autologous platelets: 11.79 ± 2.07 days No significantly different results (0/1)	Low
	Cordoba‐Fernandez and Lobo‐Martin [26]	Nonadherent dressing and cotton gauze (20)	Conventional gelatin sponges (25)	Time for full healing (days) Control: 15.1 ± 4.2 days Intervention 1: 15.3 ± 3.6 days Intervention 2: 16.0 ± 4.0 days No significantly different results (0/3)	Low
			High porosity gelatin sponges (29)		
	Dovison and Keenan [28]	Paraffin gauze (13)	Povidone‐iodine (13)	Time for full healing (days) Control: 34.2 ± 7.7 days Intervention 1: 34.3 ± 4.8 days Intervention 2: 33.3 ± 6.0 days No significantly different results (0/3)	High
			Hydrogel (16)		
	Foley and Allen [4]	Low adherent dressing (35)	Calcium alginate dressings (35)	Time for full healing (days) Control: 34.4 ± 15.2 days Intervention: 25.8 ± 12.9 days Significantly different results (1/1) Calcium alginate dressings reduce average healing time compared to low adherent dressings (*p* < 0.05)	High
	Lopezosa‐Reca et al. [30]	Povidone iodine gel (35)	2% hyaluronic acid (35)	Time for full healing (days) Control: 26.17 ± 7.75 days Intervention: 22.42 ± 2.41 days Significantly different results (1/1) Hyaluronic acid reduced average healing time compared to povidone‐iodine gel (*p* = 0.007)	Medium
	McIntosh and Thomson [31]	Paraffin tulle gras (48)	Honey dressing (52)	Time for full healing (days) Control: 39.98 ± 25.42 days Intervention: 40.3 ± 18.21 days No significantly different results (0/1)	Medium
	van Gils et al. [34]	Sulfadiazine silver cream and gauze (10)	Collagen alginate (9)	Time for full healing (days) Control: Mean 35.8 (range 19–56) days Intervention: Mean 24.4 (range 14–35) days Significantly different results (1/1) Collagen alginate reduces average healing time compared to sulfadiazine silver (*p* = 0.03)	High
Pain	Altman et al. [23]	Gauze and Burow's solution (39)	Silver sulfadiazine, gauze and Burow's solution (66)	Reduction in patients reporting any pain from week 1 to Week 4 Control: 71.8%–12.8% Intervention 1: 44%–3% Intervention 2: 55%–5% Intervention 3: 39%–1% (No significance values given)	High
			Hydrocortisone, gauze and Burow's solution (20)		
			Silver sulfadiazine, 1% hydrocortisone, gauze and Burow's solution (106)		
	Bernardshaw et al. [25]	Paraffin gauze (32)	Acidic soap (34)	Intervention 1 versus control: OR for presence of pain (at 1 week) = 3.9 (0.1–15.1) (*p* < 0.05) Intervention 2 versus control: OR for presence of pain (at 1 week) = 4.5 (1.1–18.4) (*p* = 0.04) Intervention 1 versus control: OR for presence of pain (at 2 weeks) = 2.4 (0.6–9.5) Intervention 2 versus control: OR for presence of pain (at 2 weeks) = 1.7 (0.4–7.3) Significantly different results (2/4) Acidic and alkaline soap baths had significantly increased odds of pain being present at 1 week compared to paraffin gauze	Medium
			Alkaline soap (31)		
	Cordoba‐Fernandez et al. [27]	Nitrofurazone ointment (35)	Autologous platelets (35)	Patient reported pain using the analogue chromatic visual scale Control: Postoperative day 1: 5.0 ± 2.24 cm Postoperative day 2: 3.4 ± 2.22 cm Postoperative day 3: 2.8 ± 2.16 cm Intervention: Postoperative day 1: 4.2 ± 2.22 cm Postoperative day 2: 3.0 ± 1.79 cm Postoperative day 3: 2.1 ± 1.55 cm No significantly different results (0/3)	Low
	Cordoba‐Fernandez and Lobo‐Martin [26]	Non‐adherent dressing and cotton gauze (20)	Conventional gelatin sponges (25)	Patient reported pain using the VAS Control (0–10): Postoperative day 1: 4.2 ± 1.9 cm Postoperative day 2: 2.4 ± 2.1 cm Postoperative day 3: 0.6 ± 1.1 cm Intervention 1 (0–10): Postoperative day 1: 5.0 ± 2.3 cm Postoperative day 2: 3.6 ± 2.7 cm Postoperative day 3: 1.0 ± 1.6 cm Intervention 2 (0–10): Postoperative day 1: 4.2 ± 2.5 cm Postoperative day 2: 2.3 ± 2.4 cm Postoperative day 3: 0.9 ± 2.0 cm No significantly different results (0/6)	Low
			High porosity gelatin sponges (29)		
	Drago et al. [29]	Otic corticosporin (6) Silvadene (6) Domeboro (3) Saline (2) or neosporin powder (5)	Dextran (14)	Postoperative pain on a scale of 0–3 Control: 0.57 Intervention: 0.57 (No significance values given)	High
	Lopezosa‐Reca et al. [30]	Povidone iodine gel (35)	2% hyaluronic acid (35)	Postoperative pain (VAS) Control: Time 1: 3.08 ± 1.61 cm Time 2: 1.94 ± 0.92 cm Time 3: 0.47 ± 1.16 cm Time 4: 0.14 ± 0.49 cm Intervention: Time 1: 2.42 ± 1.20 cm Time 2: 2.42 ± 1.27 cm Time 3: 0.33 ± 0.79 cm Time 4: 0.19 ± 0.47 cm No significantly different results (0/4)	Medium
	McIntosh and Thomson [31]	Paraffin tulle gras (48)	Honey dressing (52)	Postoperative pain (VAS) Control: 1.57 ± 1.3 cm Intervention: 1.6 ± 1.22 cm No significantly different results (0/1)	Medium
	Oliveira et al. [32]	Petroleum jelly and gauze (11)	Cellulose nail (15 patients)	Postoperative pain (VAS) Control (0–10): 2 days: 6.00 ± 3.34 cm 7 days: 4.18 ± 2.75 cm 15 days: 2.73 1.85 cm 30 days: 0.36 ± 0.81 cm 90 days: 0 cm 180 days: 0 cm Intervention (0–10): 2 days: 3.40 ± 1.18 cm 7 days: 2.27 ± 1.03 cm 15 days: 1.33 ± 0.49 cm 30 days: 1.07 ± 0.26 cm 90 days: 0.93 ± 0.26 cm 180 days: 1.00 ± 0.00 cm Significantly different results (1/6) Cellulose nail dressings reduce the average pain at day 15 when compared to petroleum jelly (*p* = 0.003)	Medium
Exudate (time)	Aksakal et al. [22]	Dry sterile sponge (25)	Ferric chloride (32)	Average exudation time (days) Control: 26.37 ± 7.11 days Intervention: 6.16 ± 6.53 days Significantly different results (1/1) Ferric chloride reduced period of wound exudation when compared to no topical medication (*p* = 0.000)	High
	Altman et al. [23]	Gauze and Burow's solution (39)	Silver sulfadiazine, gauze and Burow's solution (66)	Reduction in presence of exudate from week 1 to Week 4 Control: 66.7%–21% Intervention 1: 71.2%–9.1% Intervention 2: 90%–15% Intervention 3: 54%–2% (No significance values given)	High
			Hydrocortisone, gauze and Burow's solution (20)		
			Silver sulfadiazine, 1% hydrocortisone, gauze and Burow's solution (106)		
	Drago et al. [29]	Otic corticosporin (6) Silvadene (6) Domeboro (3) Saline (2) or neosporin powder (5)	Dextran (14)	Average exudation time (days) Control: 10 days Intervention: 14.7 days (No significance values given)	High
	Lopezosa‐Reca et al. [30]	Povidone iodine gel (35)	2% hyaluronic acid (35)	Number of patients with signs of exudate. Control (Yes:No): Time 1: 33:2 Time 2: 15:20 Time 3: 35:0 Time 4: 35:0 Intervention (Yes:No): Time 1: 32:3 Time 2: 11:24 Time 3: 30:5 Time 4: 35:0 No significantly different results (0/4)	Medium
	Oliveira et al. [32]	Petroleum jelly and gauze (11)	Cellulose nail (15)	Percent of patients with signs of exudation. Control: 2 days: 81.8% 7 days: 72.7% 15 days: 54.5% 30 days: 9.1% 90 days: 0% 180 days: 0% Intervention: 2 days: 73.3% 7 days: 26.7% 15 days: 6.7% 30 days: 0% 90 days: 0% 180 days: 0% Significantly different results (2/6) Cellulose nail dressings reduce the number of patients showing signs of exudate when compared to petroleum jelly at 7 days (*p* = 0.045) and 15 days (*p* = 0.021)	Medium
Exudate (size)	Drago et al. [29]	Otic corticosporin (6) Silvadene (6) Domeboro (3) Saline (2) or neosporin powder (5)	Dextran (14)	Average size of exudate on the dressing Control: 2.56 mm Intervention: 2.35 mm (No significance values given)	High
Exudate (type)	Oliveira et al. [32]	Petroleum jelly and gauze (11)	Cellulose nail dressings (15 patients)	Patients with exudate (%): serous/serosanguineous/blood/purulent Control: 2 days: 0/18.2/63.6/0 7 days: 9.1/9.1/54.5/0 15 days: 18.2/9.1/27.3/0 30 days: 0/0/0/9.1 90 days: 0/0/0/0 180 days: 0/0/0/0 Intervention: 2 days: 0/33.3/40/0 (*p* = 0.568) 7 days: 6.7/13.3/6.7/0 (*p* = 0.023) 15 days: 6.7/0/0/0 (*p* = 0.011) 30 days: 0/0/0/0 (*p* = 0.423) 90 days: 0/0/0/0 (N/A) 180 days: 0/0/0/0 (N/A) Significantly different results (2/6) Cellulose nail dressings reduce the rate of sanguineous, serosanguinous and purulent exudation at days 7 and 15	Medium
Inflammation	Altman et al. [23]	Gauze and Burow's solution (39)	Silver sulfadiazine, gauze and Burow's solution (66)	Reduction in signs of inflammation from week 1 to Week 4 Control: 74.4%–15% Intervention 1: 51.1%–3% Intervention 2: 70%–10% Intervention 3: 28%–2% (No significance values given)	High
			Hydrocortisone, gauze and Burow's solution (20)		
			Silver sulfadiazine, 1% hydrocortisone, gauze and Burow's solution (106)		
	Cordoba‐Fernandez et al. [27]	Nitrofurazone ointment (35)	Autologous platelets (35)	Average increase in toe circumference (%) Control: 6.47 ± 3.91 Intervention: 7.47 ± 3.54 No significantly different results (0/1)	Low
	Cordoba‐Fernandez and Lobo‐Martin [26]	Nonadherent dressing and cotton gauze (20)	Conventional gelatin sponges (25)	Average increase in toe circumference (cm) Control: 8.8 ± 0.6 cm Intervention 1: 8.8 ± 0.7 cm Intervention 2: 8.8 ± 0.6 cm No significantly different results (0/3)	Low
			High porosity gelatin sponges (29)		
	Lopezosa‐Reca et al. [30]	Povidone iodine gel (35)	2% hyaluronic acid (35)	Number of patients who show signs of inflammation Control (Yes:No): Time 1: 34:1 Time 2: 6:29 Time 3: 35:0 Time 4: 33:2 Intervention (Yes:No): Time 1: 33:2 Time 2: 4:31 Time 3: 28:7 Time 4: 6:29 No significantly different results (0/4)	Medium
Infection	Altman et al. [23]	Gauze and Burow's solution (39)	Silver sulfadiazine, gauze and Burow's solution (66)	Presence of infection week 1 to Week 2 Control: 3–0 Intervention 1: 2–0 Intervention 2: 20–10 Intervention 3: 2–0 (No significance values given)	High
			1% hydrocortisone, gauze and Burow's solution (20)		
			Silver sulfadiazine, 1% hydrocortisone, gauze and Burow's solution (106)		
	Bernardshaw et al. [25]	Paraffin gauze (32)	Acidic soap (34)	Intervention 1 versus control: OR for presence of infection (at 1 week) = 6.7 (1.8–24.9) (*p* = 0.005) Intervention 2 versus control: OR for presence of infection (at 1 week) = 6.0 (1.6–23.3) (*p* = 0.009) Intervention 1 versus control: OR for presence of infection (at 2 weeks) = 0.6 (0.2–2.2) Intervention 2 versus control: OR for presence of infection (at 2 weeks) = 1.5 (0.4–5.3) Significantly different results (2/4) Acidic and alkaline soap baths had significantly increased odds of infection being present at 1 week compared to paraffin gauze.	Medium
			Alkaline soap (31)		
	McIntosh and Thomson [31]	Paraffin tulle gras (48)	Honey dressing (52)	Presence of infection (no. of patients) Control: 1 Intervention: 0 No significantly different results (0/1)	Medium
Bleeding	Cordoba‐Fernadez and Lobo‐Martin [26]	Nonadherent dressing and cotton gauze (20)	Conventional gelatin sponges (25)	Weight of blood caught in the dressing Control: 3.5 ± 2.3 g Intervention 1: 1.9 ± 0.8 g Intervention 2: 1.9 ± 0.8 g Significantly different results (2/3) Conventional gelatin sponges and high porosity gelatin sponges significantly decreased post‐surgical bleeding compared to non‐adherent dressing (0.005)	Low
			High porosity gelatin sponges (29)		
Function	Bernardshaw et al. [25]	Paraffin gauze (32)	Acidic soap (34)	Intervention 1 versus control: OR for reduced function (at 1 week) = 0.7 (0.2–2.8) Intervention 2 versus control: OR for reduced function (at 1 week) = 0.6 (0.1–2.6) Intervention 1 versus control: OR for reduced function (at 2 weeks) = 0.9 (0.1–5.5) Intervention 2 versus control: OR for reduced function (at 2 weeks) = 1.1 (0.2–7.3) No significantly different results (0/4)	Medium
			Alkaline soap (31)		
Soothing effect	Bernardshaw et al. [25]	Paraffin gauze (32)	Acidic soap (34)	Specific numbers not reported. Fewer patients in the control group reported the treatment was soothing but this was no statistically significant (*p* = 0.177) No significantly different results (0/2)	Medium
			Alkaline soap (31)		
Nail bed area preservation	Oliveira et al. [32]	Petroleum jelly and gauze (11)	Cellulose nail dressings (15)	Area of nail bed conserved after treatment Control: 90.23 ± 22.53 mm^2^ (at 180 days) Intervention: 128.95 ± 49.40 mm^2^ (at 180 days) Significantly different results (1/1) Cellulose nail dressings increase average nail bed area at day 180 compared to petroleum jelly (*p* = 0.024)	Medium
Normal shoe wear	Drago et al. [29]	Otic corticosporin (6) Silvadene (6) Domeboro (3) Saline (2) or neosporin powder (5)	Dextran (14)	Days before able to wear shoes Control: 5.1 days Intervention: 4.1 days (No significance values given)	High
Satisfaction rate	Oliveira et al. [32]	Petroleum jelly and gauze (11)	Cellulose nail dressings (15)	Patient satisfaction (%): Yes/indifferent/no Control: 2 days: 27/63.6/9.1 7 days: 63.6/36.4/0 15 days: 81.8/18.2/0 Intervention: 2 days: 66.7/33.3/0 7 days: 93.3/6.7/0 15 days: 100/0/0 No significantly different results (0/3)	Medium
Ease of removal by dressing	Sykes et al. [33]	N/A	Light paraffin (93)	% of nurses who reported ‘easy removal’: Light paraffin: 73% Heavy paraffin: 90% Low adherent dressing: 63% (No significance values given)	High
			Heavy paraffin (93)		
			Low adherent dressing (88)		
Ease of dressing removal by surgery type	Sykes et al. [33]	N/A	TNA phenol (64)	% of nurses who reported ‘easy removal’: TNA phenol: 69% Zadik's procedure: 37% PNA phenol: 81% Nail plate avulsion: 64% (No significance values given)	High
			Zadik (11)		
			PNA phenol (178)		
			Surgical avulsion (21)		

Abbreviations: N/A, not applicable; NRS, numerical pain rating scale; OR, odds ratio; PNA, partial nail avulsion; TNA, total nail avulsion; VAS, visual analogue pain scale.

#### 3.4.1 Healing Rate

Three RCTs reported faster healing with calcium alginate [4], hyaluronic acid [30] and calcium alginate [34], although each had medium to high risk of bias. Four RCTS showed no advantage for autologous platelets [27], gelatin sponges [26], povidone‐iodine or hydrogel [28] and honey [31]. A small case series suggested rapid healing with oakin hydrogel (high risk of bias) [24]. Overall, evidence is inconclusive for which dressing regimen is the best for reducing healing time.

#### 3.4.2 Pain

Eight studies assessed postoperative pain (seven RCTs and one quasi‐experimental study). Alkali/acid baths increased pain initially in the first week but then subsided in the second week [25]. Cellulose nail dressings decreased the average level of pain at 15 days [32]. Both articles had a medium risk of bias. No significant benefits were found in the RCTs investigating autologous platelets [27], conventional gelatin sponges [26], high porosity gelatin sponges [26], hyaluronic acid [30], honey dressings [31] and dextran [29]. Risk of bias ranged from low to high across these RCTs. The quasi‐experimental study by Altman et al. [23] found silver sulfadiazine reduced reported pain levels from 44% to 3%, hydrocortisone from 55% to 5%, and the combination of silver sulfadiazine and hydrocortisone reduced pain from 39% to 1%. However, this study had a high risk of bias, Overall, certainty of which dressing regimen can reduce pain is inconclusive.

#### 3.4.3 Exudate

Two RCTs investigating hyaluronic acid [30] and cellulose dressings [32] and one quasi‐experimental study investigating ferric chloride [22] found significant differences in duration of exudate. The RCTS were medium risk of bias; however, the quasi‐experimental study was high risk of bias. The remaining quasi‐experimental study by Altman et al. [23] investigated silver sulfadiazine, hydrocortisone and a combination of both sulfadiazine and hydrocortisone and found that all three interventions reduced the number of patients that showed signs of exudate by the end of the trial, with silver sulfadiazine reducing the number of patients that showed signs of exudate from 54%–90% to 2%–15%. However, this study had a high risk of bias. Conversely, Drago et al. [29] found dextran increased the average duration of exudate from 10 to 14.7 days. Drago also found that dextran had a small impact on exudate diameter. However, this study did not give significance values and was at high risk of bias. The RCT by Oliveira et al. [32] also investigated the exudate type and found that cellulose dressings reduced the proportion of patients exhibiting signs of sanguineous, serosanguineous and purulent exudate. Although findings show promise for certain dressing reducing exudate, the results should be viewed cautiously due to risk of bias in the studies.

#### 3.4.4 Inflammation

None of the three RCTs detected differences in inflammation between autologous platelets [27], conventional or high‐porosity gelatin sponges [26] or hyaluronic acid [30]. The risk of bias of the RCTS was from low [26, 27] to medium [30]. One quasi‐experimental study [23] found examined silver sulfadiazine, hydrocortisone and a combination of silver sulfadiazine and hydrocortisone all reduced inflammation. However, significance values were not reported, and the study had a high risk of bias. Overall, no intervention showed clear anti‐inflammatory benefit.

#### 3.4.5 Infection

One RCT with a medium risk of bias reported a transient rise in infection with alkali/acid baths [25]. An RCT investigating honey dressings [31] and a quasi‐experimental study investigating silver sulfadiazine, hydrocortisone and a combination of the two [23] found no significant effect on infection. These studies had a medium risk of bias and a high risk of bias, respectively. No dressing investigated consistently prevented infection.

#### 3.4.6 Bleeding

One low risk of bias RCT demonstrated that conventional and high‐porosity gelatin sponges both reduced postoperative bleeding weight compared to a control [26].

#### 3.4.7 Miscellaneous

Cellulose dressings preserved a statistically significant greater nail bed area compared to petroleum jelly and gauze [32]. No significant effects were observed for postoperative function or soothing effect [25], time to normal shoe wear [29], patient satisfaction [32] or ease of dressing removal [33].

## 4 Discussion

This scoping review mapped 27 dressing regimens reported across 14 studies and 15 outcome measures. No study used a validated outcome instrument, and most relied on author‐defined metrics that varied widely across trials. Methodological quality was generally poor: eight studies were assessed as high risk of bias, four as medium and only two as low. Given this heterogeneity and pervasive bias, the current evidence base is insufficient to recommend any specific postoperative dressing for toenail surgery.

Most trials evaluated a primary dressing in isolation, seldom specifying whether a secondary dressing was applied. This focus on the primary dressing reflects the assumption that direct wound contact exerts the greatest therapeutic effect. None of the included studies used an undressed (open wound) control. This was likely due to the heightened infection risk associated with exposed foot wounds. Comparator arms were inconsistent: roughly half contrasted an ‘active’ dressing with a passive occlusive dressing (e.g., [4, 31, 32]), whereas others compared two active dressings (e.g., [27, 30]). The absence of a standardised control limits cross‐study comparability and hampers translation to clinical practice.

### 4.1 Infection Rate

Postoperative infection remains the leading complication of toenail surgery because periungual skin cannot be fully sterilised [27]. Despite antimicrobial claims for many dressings [35], only three of the 14 studies quantified infection outcomes [23, 25, 31] and two others merely noted infection events in the intervention groups [22, 28]. Small samples were a major barrier, where achieving 80% power to detect a 50% reduction in infections would require > 1000 participants [36], whereas the largest trial enrolled just 231 [23].

Comparability across studies was further undermined by the absence of a validated definition of ‘infection’ [13]. Two trials provided no criteria [23, 31]; the third used ‘redness, swelling, oedema’ [25], which are signs that phenol itself can produce [37, 38]. None reported raw infection counts or controlled for comorbidities that influence immunity, making synthesis impossible.

In short, evidence is too sparse and heterogeneous to identify any dressing that reliably lowers infection risk after toenail surgery. Robust, adequately powered trials employing standardised infection criteria are needed.

### 4.2 Healing Rate

Eight studies assessed healing time after toenail surgery [4, 24, 26, 27, 28, 30, 31, 34]. Comparisons were impossible because ’healing’ was defined inconsistently. Six trials provided a definition, but some required re‐epithelialisation (proliferation phase) whereas others accepted minimal exudate/absence of redness (late‐inflammation phase) [39]. Two papers gave no definition [24, 31].

Healing also varied by surgical modality. Phenol matrixectomies predominated, but trials included sodium‐hydroxide matrixectomies [34] and the Suppan I primary‐closure technique [27]. Wounds healing by primary intention close faster than secondary‐intention cautery wounds [40]. Similarly, NaOH heals more rapidly than phenol [41]. Even within phenol studies, application time ranged from 60 to 90 s and was unreported in two studies. This made comparison difficult as longer exposure slows healing [13] because phenol penetrates until protein coagulation halts diffusion [42].

Only two high‐bias RCTs from the 1990s showed faster healing with alginate dressings [4, 34] and have never been replicated. Given the heterogeneous definitions, differing surgical techniques and phenol protocols and the overall high risk of bias, no dressing regimen can presently be recommended to accelerate healing after toenail surgery.

### 4.3 Exudate

Exudation was evaluated in five studies, four of which followed phenol matrixectomy [22, 23, 29, 30, 32]. Of the three trials reporting significant effects, two demonstrated only transient reductions: hyaluronic‐acid dressings [30] and cellulose dressings [32]. Only one study showed a sustained benefit of ferric chloride shortening exudation from approximately 26 to 6 days [22]. However, this study carried a high risk of bias. Overall, evidence is insufficient to identify any dressing that reliably limits postoperative exudate.

### 4.4 Pain

Similarly to the other outcome measures, the measurement of pain in included articles was substantially varied. Methods of assessing pain included absence of pain [23], VAS [25, 26, 27, 30, 31, 32], NRS [25] and a scale of 0–3 [29]. None of the included articles showed a nontransient statistically significant result.

Although no article presented a potential mechanism of action for their chosen dressing regimen to reduce pain, the measurement of pain may have been used to track pain as a confounding factor for other outcome measures. For example, Bernardshaw et al. [25] measured the response to pain as it was a confounding factor for the soothing effect of the dressing regimen assessed. The articles by Lopezosa‐Reca et al. [30] and McIntosh and Thomson [31] investigated both healing rate and pain. Pain is a known factor in reducing the rate of healing [43], as the mediators which create the nociception signal also affect the release of a multitude of physiological compounds which are vital for wound healing [44]. Therefore, pain may have been measured to assess the effect it had on their reported healing rate. Overall, no dressing regimen was found to be superior in reducing pain post‐toenail surgery.

### 4.5 Other Outcome Measures

Cordoba‐Fernadez and Lobo‐Martin [26] found that both high porosity and conventional gelatin sponges reduced the average weight of postoperative bleeding. However, the distinction between bleeding and other types of exudate was unclear. They conducted phenolisation surgeries which may increase the levels of exudation and consequently there may be more exudate in the dressings than expected which may be a confounding factor.

Both function [25] and time for normal shoe wear [29] may be interpreted as substitutes for healing time as both are aspects of a return to normal daily activities. Neither study found a significant difference in the investigated interventions. Therefore, no conclusions can be made on which dressing may get patients into normal daily activities in a timelier manner.

### 4.6 Limitations

The limitations of this review include the limited studies available on certain cautery agents, the high risk of bias of included papers, the chosen risk of bias cutoffs, the low number of included studies and the limited available studies on different dressing regimens.

Other limitations of this review include that the searches were limited to English only due to the languages limitations of the reviewers, the limited number of databases used to conduct the search and the potential lack of applicability of this review due to the heterogeneity of included articles.

### 4.7 Further Research

This review highlights the need for standardised definitions of infection and healing and the standardisation of outcome measures in toenail surgeries to allow the comparison of current dressings and any dressings developed in the future. Many surgical modalities (e.g., laser, cryotherapy and electrocautery) have not been evaluated, and therefore, further research is needed to understand the subsequent wound physiology.

### 4.8 Conclusion

Extensive variability in the types of dressing being utilised in toenail surgeries and variability in the outcome measures used to investigate them meant collation of results for similar outcome measures, primarily healing rate and postoperative complications, was not possible, and the clinical conclusions reached were limited. However, some different dressing options that may improve postoperative outcomes were identified, with the consensus from included studies being that using a primary dressing was more likely to have greatest therapeutic effect compared to secondary dressings. Further high‐quality studies with standardised outcome measures are needed to determine the most efficacious postoperative dressing regimen following toenail surgery.

## Author Contributions

**David Yousry Foad Isaac Wassef:** methodology, data curation, formal analysis, writing – original draft, writing – review and editing. **Zainab Al‐Modhefer:** conceptualisation, methodology, data curation, supervision, writing – original draft, writing – review and editing. **Kym Hennessy:** conceptualisation, methodology, data curation, formal analysis, supervision, writing – original draft, writing – review and editing.

## Funding

The authors have nothing to report.

## Conflicts of Interest

The authors declare no conflicts of interest.

## Supporting information


Supporting Information S1

## Data Availability

All data generated and analysed for this study are available in this scoping review or the original studies included.
