# Supplementary material for: Postoperative Dressing Regimens in Nail Surgeries: A Scoping Review
Source: J Foot Ankle Res. 2026 Jan 21;19(1):e70100. doi: 10.1002/jfa2.70100 (PMC12823457; doi:10.1002/jfa2.70100)
Supplement: Supplementary file 1 — Supporting Information S1 [file JFA2-19-e70100-s001.docx]

Additional File A: Detailed Search Strategy

| Table 1 Medline and Embase search strategy | |
| --- | --- |
| Search ID | Search string |
| 1 | (onychocryptosis or ingrown toenail* or ingrown nail* or toenail or onychomycosis or Nail Disease).ti,ab. |
| 2 | Nails, Ingrown/ or Nail Diseases/ or Onychomycosis/ |
| 3 | 1 or 2 |
| 4 | (Matri?ectom* or Toenail Avulsion* or Nail Avulsion* or Nail Removal or Nail Excision* or Nail Surgery or Phenol* or Surgical Wound or Foot Surgery).ti,ab. |
| 5 | foot surgery/ or surgical wound/ or nail/su [Surgery] |
| 6 | 4 or 5 |
| 7 | (Wound Healing or Dressing* or Bandage* or Postoperative Care or Postoperative or Surgical Wound Care).ti,ab. |
| 8 | wound dressing/ or bandage/ or postoperative care/ or wound healing/ or wound care/ |
| 9 | 7 or 8 |
| 10 | 3 and 6 and 9 |

| *Table 2 CINAHL search strategy* | |
| --- | --- |
| Search ID | Search string |
| S1 | TI ( onychocryptosis OR "ingrown toenail*" OR "ingrown nail*" OR toenail* OR onychomycosis OR “Nail Disease” ) OR AB ( onychocryptosis OR "ingrown toenail*" OR "ingrown nail*" OR toenail* OR onychomycosis OR “Nail Disease” ) |
| S2 | (MH "Nails, Ingrown") OR (MH "Nail Diseases") OR (MH "Onychomycosis") |
| S3 | S1 OR S2 |
| S4 | TI (Matri?ectom* OR “Toenail Avulsion*” OR “Nail Avulsion*” OR “Nail Removal*” OR “Nail Excision*” OR "Toenail Surger*" OR "Nail Surger*" OR Phenol* OR “Surgical Wound” OR "Foot surger*") OR AB (Matri?ectom* OR “Toenail Avulsion*” OR “Nail Avulsion*” OR “Nail Removal*” OR “Nail Excision*” OR "Nail Surger*" OR Phenol* OR “Surgical Wound” OR "Foot surger*") |
| S5 | (MH "Nails/SU") OR (MH "Surgery, Podiatric") OR MH ("Surgical Wound") |
| S6 | S4 OR S5 |
| S7 | TI (“Wound Healing” OR Dress* OR Bandage* OR “Postoperative Care” OR Postoperative OR “Surgical Wound Care”) OR AB (“Wound Healing” OR Dress* OR Bandage* OR “Postoperative Care” OR Postoperative OR “Surgical Wound Care”) |
| S8 | (MH "Bandages and Dressings") OR (MH "Postoperative Care") OR (MH "Wound Healing") OR (MH "Surgical Wound Care") |
| S9 | S7 OR S8 |
| S10 | S3 AND S6 AND S9 |

*Scopus search strategy:*

( TITLE-ABS ( onychocryptosis OR "ingrown toenail" OR "ingrown nail" OR toenail OR onychomycosis OR "Nail Disease" ) ) AND ( TITLE-ABS ( matrixectom* OR “Nail Surgery” OR "Toenail Avulsion*" OR "Nail Avulsion*" OR "Nail Removal*" OR "Nail Excision*" OR phenol* OR "Surgical Wound*" OR “Toenail Surgery” OR “Foot Surgery”) ) AND ( TITLE-ABS ( "Wound Healing" OR "Dress*" OR "Bandage*" OR "Postoperative Care" OR postoperative OR "Surgical Wound Care" ) )

| *Table 3 Cochrane Library search strategy* | |
| --- | --- |
| Search ID | Search string |
| #1 | (onychocryptosis OR (ingrown NEXT toenail*) OR (ingrown NEXT nail*) OR toenail* OR onychomycosis OR "Nail Disease"):ti OR (onychocryptosis OR (ingrown NEXT toenail*) OR (ingrown NEXT nail*) OR toenail* OR onychomycosis OR "Nail Disease"):ab (Word variations have been searched) |
| #2 | MeSH descriptor: [Nail Diseases] this term only |
| #3 | MeSH descriptor: [Nails, Ingrown] this term only |
| #4 | MeSH descriptor: [Onychomycosis] this term only |
| #5 | #1 OR #2 OR #3 OR #4 |
| #6 | (Matri?ectom* OR (Toenail NEXT Avulsion*) OR (Nail NEXT Avulsion*) OR (Nail NEXT Removal*) OR (Nail NEXT Excision*) OR (Toenail NEXT Surger*) OR (Nail NEXT Surger*) OR Phenol* OR "Surgical Wound" OR (Foot NEXTSurger*)):ti OR (Matri?ectom* OR (Toenail NEXT Avulsion*) OR (Nail NEXT Avulsion*) OR (Nail NEXT Removal*) OR (Nail NEXT Excision*) OR (Toenail NEXT Surger*) OR (Nail NEXT Surger*) OR Phenol* OR "Surgical Wound" OR (Foot NEXTSurger*)):ab (Word variations have been searched) |
| #7 | MeSH descriptor: [Surgical Wound] this term only |
| #8 | MeSH descriptor: [Nails] explode all trees and with qualifier(s): [surgery - SU] |
| #9 | #6 OR #7 OR #8 |
| #10 | ("Wound Healing" OR Dress* OR Bandage* OR "Postoperative Care" OR Postoperative OR "Surgical Wound Care"):ti OR ("Wound Healing" OR Dress* OR Bandage* OR "Postoperative Care" OR Postoperative OR "Surgical Wound Care"):ab |
| #11 | MeSH descriptor: [Wound Healing] this term only |
| #12 | MeSH descriptor: [Bandages] this term only |
| #13 | MeSH descriptor: [Postoperative Care] this term only |
| #14 | #10 OR #11 OR #12 OR #13 |
| #15 | #5 AND #9 AND #14 |

**Additional File B: Critical appraisal using the Johanna Briggs Institute (JBI) tools**

| Table 1 Critical appraisal of included articles using the Johanna Briggs Institute (JBI) case series appraisal tool | | | | | | | | | | | |
| --- | --- | --- | --- | --- | --- | --- | --- | --- | --- | --- | --- |
| **JBI case series appraisal tool** | | | | | | | | | | | |
| **Study** | **Q1** | **Q2** | **Q3** | **Q4** | **Q5** | **Q6** | **Q7** | **Q8** | **Q9** | **Q10** | **% Yes** |
| Barreiro et al. (2014) | Y | N | U | U | N | N | N | N | N | U | 10% |
| *Note.* Questions adapted from Munn et al. (2020). Y = yes, N = no, U = unclear. Questions were as follows: 1. Were there clear criteria for inclusion in the case series? 2. Was the condition measured in a standard, reliable way for all participants included in the case series? 3. Were valid methods used for identification of the condition for all participants included in the case series? 4. Did the case series have consecutive inclusion of participants? 5. Did the case series have complete inclusion of participants? 6. Was there clear reporting of the demographics of the participants in the study? 7. Was there clear reporting of clinical information of the participants? 8. Were the outcomes or follow up results of cases clearly reported? 9. Was there clear reporting of the presenting site(s)/clinic(s) demographic information? 10. Was statistical analysis appropriate? | | | | | | | | | | | |

| Table 2 Critical appraisal of included articles using the Johanna Briggs Institute (JBI) quasi-experimental critical appraisal tool | | | | | | | | | | | | |
| --- | --- | --- | --- | --- | --- | --- | --- | --- | --- | --- | --- | --- |
| **JBI quasi-experimental critical appraisal tool** | | | | | | | | | | | | |
|  | **Domain** | | **Temporal precedence** | **Selection and allocation** | **Confounding factors** | **Administration of intervention / exposure** | **Assessment, detection, and measurement of the outcome** | | | **Participant retention** | **Statistical conclusion validity** | **% Yes** |
|  | **Question no.** | | **Q1** | **Q2** | **Q3** | **Q4** | **Q5** | **Q6** | **Q7** | **Q8** | **Q9** |  |
| **Study** | **Outcome** | **Result** |  |  |  |  |  |  |  |  |  |  |
| Aksakal et al. (2001) | Exudate | Duration | Y | U | Y | Y | N/A | Y | U | N | N | 44% |
| Altman et al. (1990) | Pain | Week 1 | Y | U | Y | Y | N/A | Y | U | U | N | 44% |
|  | Pain | Week 2 |  |  |  |  |  |  |  | U | N |  |
|  | Pain | Week 3 |  |  |  |  |  |  |  | U | N |  |
|  | Pain | Week 4 |  |  |  |  |  |  |  | U | N |  |
|  | Drainage | Week 1 |  |  |  |  | N/A | Y | U | U | N | 44% |
|  | Drainage | Week 2 |  |  |  |  |  |  |  | U | N |  |
|  | Drainage | Week 3 |  |  |  |  |  |  |  | U | N |  |
|  | Drainage | Week 4 |  |  |  |  |  |  |  | U | N |  |
|  | Inflammation | Week 1 |  |  |  |  | N/A | Y | U | U | N | 44% |
|  | Inflammation | Week 2 |  |  |  |  |  |  |  | U | N |  |
|  | Inflammation | Week 3 |  |  |  |  |  |  |  | U | N |  |
|  | Inflammation | Week 4 |  |  |  |  |  |  |  | U | N |  |
|  | Infection | Week 1 |  |  |  |  | N/A | Y | U | U | N | 44% |
|  | Infection | Week 2 |  |  |  |  |  |  |  | U | N |  |
|  | Infection | Week 3 |  |  |  |  |  |  |  | U | N |  |
|  | Infection | Week 4 |  |  |  |  |  |  |  | U | N |  |
| *Note.* Y = yes, N = no, U = unclear, N/A = not applicable. Questions adapted from Barker et al., (2024). Questions were as follows: 1. Is it clear in the study what is the “cause” and what is the “effect”? 2. Was there a control group? 3. Were participants included in any comparisons similar? 4. Were the participants included in any comparisons receiving similar treatment/care, other than the exposure or intervention of interest? 5. Were there multiple measurements of the outcome, both pre and post the intervention/exposure? 6. Were the outcomes of participants included in any comparisons measured in the same way? 7.Were outcomes measured in a reliable way? 8.Was follow-up complete and if not, were differences between groups in terms of their follow-up adequately described and analysed? 9. Was appropriate statistical analysis used? | | | | | | | | | | | | |

| Table 3 Critical appraisal of included articles using the Johanna Briggs Institute (JBI) randomised control trial (RCT) critical appraisal tool | | | | | | | | | | | | | | | | |
| --- | --- | --- | --- | --- | --- | --- | --- | --- | --- | --- | --- | --- | --- | --- | --- | --- |
| **JBI RCT critical appraisal tool** | | | | | | | | | | | | | | | | |
|  | | **Domain** | **Selection and allocation** | | | **Administration of intervention/ exposure** | | | **Assessment, detection, and measurement of the outcome** | | | **Participant retention** | **Statistical conclusion validity** | | | **%Yes** |
|  |  | **Question number** | **Q1** | **Q2** | **Q3** | **Q4** | **Q5** | **Q6** | **Q7** | **Q8** | **Q9** | **Q10** | **Q11** | **Q12** | **Q13** |  |
| **Study** | **Outcome** | **Result** |  |  |  |  |  |  |  |  |  |  |  |  |  |  |
| Bernardshaw et al. (2019) | Pain | Baseline | Y | Y | U | N | N | Y | Y | Y | U | Y | N | Y | Y | 62% |
|  | Pain | Week 1 |  |  |  |  |  |  |  |  |  | Y | N | Y |  |  |
|  | Pain | Week 2 |  |  |  |  |  |  |  |  |  | Y | N | Y |  |  |
|  | Function | Baseline |  |  |  |  |  |  | Y | Y | U | Y | N | Y |  | 62% |
|  | Function | Week 1 |  |  |  |  |  |  |  |  |  | Y | N | Y |  |  |
|  | Function | Week 2 |  |  |  |  |  |  |  |  |  | Y | N | Y |  |  |
|  | Infection | Baseline |  |  |  |  |  |  | U | N | U | Y | N | Y |  | 62% |
|  | Infection | Week 1 |  |  |  |  |  |  |  |  |  | Y | N | Y |  |  |
|  | Infection | Week 2 |  |  |  |  |  |  |  |  |  | Y | N | Y |  |  |
|  | Soothing effect | Week 1 |  |  |  |  |  |  | U | U | U | Y | N | Y |  | 62% |
|  | Soothing effect | Week 2 |  |  |  |  |  |  |  |  |  | Y | N | Y |  |  |
| Cordoba-Fernandez and Lobo-Martin (2022) | Weight of dressing (bleeding) | 48 hours post-surgery | Y | N | Y | Y | Y | Y | Y | Y | U | Y | Y | Y | Y | 77% |
|  | Pain | Day 1 |  |  |  |  |  |  | Y | Y | U | Y | Y | Y |  | 77% |
|  | Pain | Day 2 |  |  |  |  |  |  |  |  |  | Y | Y | Y |  |  |
|  | Pain | Day 3 |  |  |  |  |  |  |  |  |  | Y | Y | Y |  |  |
|  | Inflammation (digital circumference) | Pre-operatively |  |  |  |  |  |  | Y | Y | U | Y | Y | Y |  | 77% |
|  | Inflammation (digital circumference) | 48 hours postoperatively |  |  |  |  |  |  |  |  |  | Y | Y | Y |  |  |
|  | Recovery time | Fully healed |  |  |  |  |  |  | Y | Y | U | Y | Y | Y |  | 77% |
| Cordoba-Fernandez et al. (2010) | Recovery time | Fully healed | U | Y | Y | Y | N | Y | Y | Y | U | Y | Y | Y | Y | 77% |
|  | Pain | Day 1 |  |  |  |  |  |  | Y | Y | U | Y | Y | Y |  | 77% |
|  | Pain | Day 2 |  |  |  |  |  |  | Y | Y | U | Y | Y | Y |  |  |
|  | Pain | Day 3 |  |  |  |  |  |  | Y | Y | U | Y | Y | Y |  |  |
|  | Inflammation | 48 hours |  |  |  |  |  |  | Y | Y | U | Y | Y | Y |  | 77% |
| Dovison and Keenan (2001) | Recovery time | Fully healed | U | U | U | U | U | Y | U | Y | U | N | N | U | N | 15% |
| Drago et al. (1983) | Pain | Day 3 | U | U | N | N | U | Y | N | Y | U | N | N | N | N | 15% |
|  | Normal shoe wear | Days until normal shoe wear |  |  |  |  |  |  | N | Y | U | N | N | N |  | 15% |
|  | Exudate (mm) | At check-up (unknown time) |  |  |  |  |  |  | N | Y | U | N | N | N |  | 15% |
|  | Exudate (days) | Days until no exudate |  |  |  |  |  |  | N | Y | U | N | N | N |  | 15% |
| Foley and Allen (1994) | Healing time | Days for full healing | U | U | Y | U | N | Y | U | U | U | Y | Y | U | Y | 38% |
|  | Dressing changes | How many dressings for full healing |  |  |  |  |  |  | U | U | U | Y | Y | U |  | 38% |
| Lopezosa-Reca et al. (2023) | Healing time | Time until full healing | Y | U | U | U | N | Y | Y | Y | U | Y | Y | Y | Y | 62% |
|  | Pain | Time 1 |  |  |  |  |  |  | Y | Y | U | Y | Y | Y |  | 62% |
|  | Pain | Time 2 |  |  |  |  |  |  |  |  |  | Y | Y | Y |  |  |
|  | Pain | Time 3 |  |  |  |  |  |  |  |  |  | Y | Y | Y |  |  |
|  | Pain | Time 4 |  |  |  |  |  |  |  |  |  | Y | Y | Y |  |  |
|  | Inflammation | Time 1 |  |  |  |  |  |  | Y | Y | U | Y | Y | Y |  | 62% |
|  | Inflammation | Time 2 |  |  |  |  |  |  |  |  |  | Y | Y | Y |  |  |
|  | Inflammation | Time 3 |  |  |  |  |  |  |  |  |  | Y | Y | Y |  |  |
|  | Inflammation | Time 4 |  |  |  |  |  |  |  |  |  | Y | Y | Y |  |  |
|  | Exudate | Time 1 |  |  |  |  |  |  | Y | Y | U | Y | Y | Y |  | 62% |
|  | Exudate | Time 2 |  |  |  |  |  |  |  |  |  | Y | Y | Y |  |  |
|  | Exudate | Time 3 |  |  |  |  |  |  |  |  |  | Y | Y | Y |  |  |
|  | Exudate | Time 4 |  |  |  |  |  |  |  |  |  | Y | Y | Y |  |  |
| McIntosh and Thomson (2006) | Healing time | Total nail avulsion | Y | Y | N | Y | N | Y | Y | Y | U | N | Y | Y | Y | 69% |
|  | Healing time | Partial nail avulsions |  |  |  |  |  |  |  |  |  | N | Y | Y |  | 69% |
|  | Pain | First visit |  |  |  |  |  |  | Y | Y | U | N | N | Y |  | 62% |
|  | Infection | Duration of healing |  |  |  |  |  |  | Y | Y | U | N | U | Y |  | 62% |
|  | Adverse events | Duration of healing |  |  |  |  |  |  | Y | Y | U | N | U | Y |  | 62% |
| Oliveira et al. (2020) | Area | 2 days | Y | U | Y | U | N | Y | U | Y | U | Y | Y | Y | Y | 62% |
|  | Area | 180 days |  |  |  |  |  |  |  |  |  | Y | Y | Y |  |  |
|  | Pain | 2 days |  |  |  |  |  |  | U | Y | U | Y | Y | Y |  | 62% |
|  | Pain | 7 days |  |  |  |  |  |  |  |  |  | Y | Y | Y |  |  |
|  | Pain | 15 days |  |  |  |  |  |  |  |  |  | Y | Y | Y |  |  |
|  | Pain | 30 days |  |  |  |  |  |  |  |  |  | Y | Y | Y |  |  |
|  | Pain | 90 days |  |  |  |  |  |  |  |  |  | Y | Y | Y |  |  |
|  | Pain | 180 days |  |  |  |  |  |  |  |  |  | Y | Y | Y |  |  |
|  | Satisfaction rate | 2 days |  |  |  |  |  |  | U | Y | U | Y | Y | Y |  | 62% |
|  | Satisfaction rate | 7 days |  |  |  |  |  |  |  |  |  | Y | Y | Y |  |  |
|  | Satisfaction rate | 15 days |  |  |  |  |  |  |  |  |  | Y | Y | Y |  |  |
|  | Satisfaction rate | 30 days |  |  |  |  |  |  |  |  |  | Y | Y | Y |  |  |
|  | Satisfaction rate | 90 days |  |  |  |  |  |  |  |  |  | Y | Y | Y |  |  |
|  | Satisfaction rate | 180 days |  |  |  |  |  |  |  |  |  | Y | Y | Y |  |  |
|  | Wound Appearance | 2 days |  |  |  |  |  |  | U | Y | U | Y | Y | Y |  | 62% |
|  | Wound Appearance | 7 days |  |  |  |  |  |  |  |  |  | Y | Y | Y |  |  |
|  | Wound Appearance | 15 days |  |  |  |  |  |  |  |  |  | Y | Y | Y |  |  |
|  | Wound Appearance | 30 days |  |  |  |  |  |  |  |  |  | Y | Y | Y |  |  |
|  | Wound Appearance | 90 days |  |  |  |  |  |  |  |  |  | Y | Y | Y |  |  |
|  | Wound Appearance | 180 days |  |  |  |  |  |  |  |  |  | Y | Y | Y |  | 62% |
|  | Exudate type | 2 days |  |  |  |  |  |  | U | Y | U | Y | Y | Y |  | 62% |
|  | Exudate type | 7 days |  |  |  |  |  |  |  |  |  | Y | Y | Y |  |  |
|  | Exudate type | 15 days |  |  |  |  |  |  |  |  |  | Y | Y | Y |  |  |
|  | Exudate type | 30 days |  |  |  |  |  |  |  |  |  | Y | Y | Y |  |  |
|  | Exudate type | 90 days |  |  |  |  |  |  |  |  |  | Y | Y | Y |  |  |
|  | Exudate type | 180 days |  |  |  |  |  |  |  |  |  | Y | Y | Y |  |  |
|  | Exudate amount | 2 days |  |  |  |  |  |  | U | Y | U | Y | Y | Y |  | 62% |
|  | Exudate amount | 7 days |  |  |  |  |  |  |  |  |  | Y | Y | Y |  |  |
|  | Exudate amount | 15 days |  |  |  |  |  |  |  |  |  | Y | Y | Y |  |  |
|  | Exudate amount | 30 days |  |  |  |  |  |  |  |  |  | Y | Y | Y |  |  |
|  | Exudate amount | 90 days |  |  |  |  |  |  |  |  |  | Y | Y | Y |  |  |
|  | Exudate amount | 180 days |  |  |  |  |  |  |  |  |  | Y | Y | Y |  |  |
| Sykes et al. (1987) | Ease of dressing removal | Type of dressing | U | U | U | N | N | Y | N | Y | U | N | N | N | N | 15% |
|  | Ease of dressing removal | Type of operation |  |  |  |  |  |  | U | Y | U | N | N | N |  | 15% |
| van Gils et al. (1998) | Healing time | Time fully healed | U | U | U | U | N | Y | N | Y | U | N | N | Y | Y | 30% |
| *Note.*  Y = yes, N = no, U = Unclear, N/A =Not applicable, RCT = Randomised control trial. Questions adapted from Barker et al., (2023). Questions were as follows: 1. Was true randomization used for assignment of participants to treatment groups? 2. Was allocation to treatment groups concealed? 3. Were treatment groups similar at the baseline? 4. Were participants blind to treatment assignment? 5. Were those delivering the treatment blind to treatment assignment? 6. Were treatment groups treated identically other than the intervention of interest? 7. Were outcome assessors blind to treatment assignment? 8. Were outcomes measured in the same way for treatment groups? 9. Were outcomes measured in a reliable way? 10. Was follow up complete and if not, were differences between groups in terms of their follow up adequately described and analysed? 11. Were participants analysed in the groups to which they were randomized? 12. Was appropriate statistical analysis used? 13. Was the trial design appropriate and any deviations from the standard RCT design (individual randomization, parallel groups) accounted for in the conduct and analysis of the trial? | | | | | | | | | | | | | | | | |

**Additional File C**

*Preferred Reporting Items for Systematic Reviews and Meta-Analyses Scoping Review extension (PRISMA-ScR) checklist*

|  | | | |
| --- | --- | --- | --- |
| **SECTION** | **ITEM** | **PRISMA-ScR CHECKLIST ITEM** | **REPORTED ON PAGE #** |
| **TITLE** | | | |
| Title | 1 | Identify the report as a scoping review. | 1 |
| **ABSTRACT** | | | |
| Structured summary | 2 | Provide a structured summary that includes (as applicable): background, objectives, eligibility criteria, sources of evidence, charting methods, results, and conclusions that relate to the review questions and objectives. | 2-3 |
| **INTRODUCTION** | | | |
| Rationale | 3 | Describe the rationale for the review in the context of what is already known. Explain why the review questions/objectives lend themselves to a scoping review approach. | 4-5 |
| Objectives | 4 | Provide an explicit statement of the questions and objectives being addressed with reference to their key elements (e.g., population or participants, concepts, and context) or other relevant key elements used to conceptualize the review questions and/or objectives. | 5 |
| **METHODS** | | | |
| Protocol and registration | 5 | Indicate whether a review protocol exists; state if and where it can be accessed (e.g., a Web address); and if available, provide registration information, including the registration number. | 6 |
| Eligibility criteria | 6 | Specify characteristics of the sources of evidence used as eligibility criteria (e.g., years considered, language, and publication status), and provide a rationale. | 6 |
| Information sources* | 7 | Describe all information sources in the search (e.g., databases with dates of coverage and contact with authors to identify additional sources), as well as the date the most recent search was executed. | 6 |
| Search | 8 | Present the full electronic search strategy for at least 1 database, including any limits used, such that it could be repeated. | 6 |
| Selection of sources of evidence† | 9 | State the process for selecting sources of evidence (i.e., screening and eligibility) included in the scoping review. | 6-7 |
| Data charting process‡ | 10 | Describe the methods of charting data from the included sources of evidence (e.g., calibrated forms or forms that have been tested by the team before their use, and whether data charting was done independently or in duplicate) and any processes for obtaining and confirming data from investigators. | 8 |
| Data items | 11 | List and define all variables for which data were sought and any assumptions and simplifications made. | 6-7 |
| Critical appraisal of individual sources of evidence§ | 12 | If done, provide a rationale for conducting a critical appraisal of included sources of evidence; describe the methods used and how this information was used in any data synthesis (if appropriate). | 7-8 |
| Synthesis of results | 13 | Describe the methods of handling and summarizing the data that were charted. | 8 |
| **RESULTS** | | | |
| Selection of sources of evidence | 14 | Give numbers of sources of evidence screened, assessed for eligibility, and included in the review, with reasons for exclusions at each stage, ideally using a flow diagram. | 9-10 |
| Characteristics of sources of evidence | 15 | For each source of evidence, present characteristics for which data were charted and provide the citations. | 10-14 |
| Critical appraisal within sources of evidence | 16 | If done, present data on critical appraisal of included sources of evidence (see item 12). | 15 |
| Results of individual sources of evidence | 17 | For each included source of evidence, present the relevant data that were charted that relate to the review questions and objectives. | 20-32 |
| Synthesis of results | 18 | Summarize and/or present the charting results as they relate to the review questions and objectives. | 16-19 |
| **DISCUSSION** | | | |
| Summary of evidence | 19 | Summarize the main results (including an overview of concepts, themes, and types of evidence available), link to the review questions and objectives, and consider the relevance to key groups. | 33-37 |
| Limitations | 20 | Discuss the limitations of the scoping review process. | 37-38 |
| Conclusions | 21 | Provide a general interpretation of the results with respect to the review questions and objectives, as well as potential implications and/or next steps. | 38 |
| **FUNDING** | | | |
| Funding | 22 | Describe sources of funding for the included sources of evidence, as well as sources of funding for the scoping review. Describe the role of the funders of the scoping review. | 1 |
| *Note.* Table adapted from : Tricco, A. C., Lillie, E., Zarin, W., O'Brien, K. K., Colquhoun, H., Levac, D., Moher, D., Peters, M. D. J., Horsley, T., Weeks, L., Hempel, S., Akl, E. A., Chang, C., McGowan, J., Stewart, L., Hartling, L., Aldcroft, A., Wilson, M. G., Garritty, C., Lewin, S., … Straus, S. E. (2018). PRISMA Extension for Scoping Reviews (PRISMA-ScR): Checklist and Explanation. Annals of Internal Medicine, 169(7), 467–473. <https://doi.org/10.7326/M18-0850> | | | |
